# Thromboelastography-guided transfusion in dogs with hypocoagulable disorders: a case series

**DOI:** 10.1186/s13028-019-0469-x

**Published:** 2019-07-22

**Authors:** Rebecca Langhorn, Louise Bochsen, Jakob Lundgreen Willesen, Tina Møller Sørensen, Annemarie Thuri Kristensen

**Affiliations:** Department of Veterinary Clinical Sciences, Faculty of Health and Medical Sciences, University of Copenhagen, Dyrlægevej 16, 1870 Frederiksberg C, Denmark

**Keywords:** Bleeding, Dog, Hemostasis, Thromboelastography, Transfusion medicine

## Abstract

**Background:**

Thromboelastography (TEG) is a global whole blood hemostasis assay which includes plasma as well as cellular components of hemostasis in the analysis and follows the quality and dynamics of clot development, stabilization, and lysis. In human medicine TEG is also a valuable asset in the therapeutic setting, allowing evaluation of the effect of transfusion therapy in vitro. This case series describes the use of TEG as a guiding tool for transfusion therapy in four dogs with hypocoagulable hemostatic disorders.

**Case presentation:**

Four dogs presented with hypocoagulable disorders of hemostasis, diagnosed as rodenticide intoxication, angiostrongylosis, disseminated intravascular coagulation following severe systemic inflammation, and immune-mediated thrombocytopenia, respectively. TEG was used as a diagnostic tool as well as a guiding tool in the decision of whether or not, and in what dose, fresh frozen plasma would be of benefit in the treatment protocol for each dog.

**Conclusions:**

TEG may be applied in the therapeutic setting as a means to tailor individual patient transfusion therapy in critically ill dogs with hypocoagulable states.

## Background

Bleeding disorders represent a common challenge to the companion animal veterinarian with a need for thorough and timely diagnostic work-up and therapy due to the often hemodynamically unstable state of the patient. With the traditional plasma-based coagulation assays, such as prothrombin time (PT) and activated partial thromboplastin time (aPTT), only the effect of plasma coagulation factors on initiation of hemostasis is measured, and the analysis ends at the point of fibrin polymer formation. In addition, prolonged aPTT and PT do not necessarily correlate with bleeding risk [1–3]. Thromboelastography (TEG), on the other hand, is a global whole blood hemostasis assay, including plasma as well as cellular components of hemostasis and following the quality and dynamics of clot development, stabilization, and lysis [4]. Additionally, it has higher negative as well as positive predictive values for identification of patients with clinical bleeding when compared to conventional coagulation assays [1]. With the validation of the assay for use in dogs, TEG has, therefore, become a valuable diagnostic tool in the work-up of bleeding disorders in this species [5].

Interestingly, human studies have revealed that TEG may also be of value in the therapeutic setting, allowing evaluation of the effect of transfusion therapy in vitro [6]. This is a method for evaluation of the potential individual patient response to a transfusion, thereby avoiding excessive transfusion therapy and the risk of transfusion reactions in patients for whom a transfusion is not beneficial as well as optimizing the use of blood components [7].

This case series reviews four dogs with different hypocoagulable disorders and the use of TEG in guiding their therapy. The cases were seen between 2008 and 2017 at the University Hospital for Companion Animals, University of Copenhagen, and are a representative sample of the patient population for which the authors have applied TEG-guided transfusion over more than a decade. The cases presented were chosen based on two criteria: (1) Cases with different hypocoagulability disorders in order to present TEG-guided transfusion across a range of patient types (2) Cases for which a full admission data set (clinical and paraclinical results) as well as post-transfusion TEG were available.

In each case TEG analyses were performed using the computerized thromboelastograph (TEG 5000 Hemostasis Analyzer) and applying recombinant human tissue factor (TF) for activation according to a previously validated method [5]. The analysis is performed only by skilled personnel at the Veterinary Diagnostic Laboratory, University of Copenhagen, and is applied routinely in the investigation of hemostatic abnormalities at the hospital. Briefly, citrated whole blood is collected using a vacutainer system. After letting the blood rest for 30 min, it is inverted carefully 10 times, and the assay is initiated by activation of 400 µL whole blood with 25 µL diluted TF [Innovin (Dade Innovin, Siemens)]. Then, 340 µL of the TF-activated blood are transferred to a preheated (37 °C) TEG cup containing 20 µL 0.2 M CaCl_2_ and a final TF dilution of 1:50,000 [5]. The TEG assay is set to run for 2 h, however transfusion requirement can often be suspected within the first 30 min of the analysis. When evaluating the benefit of fresh frozen plasma (FFP) for a dog, a new TEG is initiated with addition of FFP in vitro (FFP-TEG). 50 µL FFP added to the 400 µL citrated whole blood corresponds to a clinical dose of 10 mL/kg FFP transfusion for a dog with a blood volume of 80 mL/kg (i.e. approximately 10%). The 340 µL sample volume of TF-activated blood for the FFP-TEG is drawn from this mixture.

The TEG parameters relevant for evaluating hypocoagulability are the reaction time (R) which reflects initiation of coagulation and relies on adequate concentrations of coagulation factors, the kinetics of coagulation reflected by the angle (α) and kinetics (K) which rely on both coagulation factors, fibrinogen and platelets, and the maximal amplitude (MA) which reflects final clot strength and depends on platelets and fibrinogen concentration [5].

## Case presentation

### Case 1

A 3-year-old 9.9 kg neutered male mixed-breed dog presented with a history of a dry cough and sudden deterioration. The referring veterinarian had suspected pneumonia based on thoracic radiographs, and the dog had been treated with antibiotics and a non-steroidal anti-inflammatory drug prior to referral. The veterinarian had also tested the dog for *Angiostrongylus vasorum* (endemic in the area), for which a fecal smear and the commercially available antigen test (IDEXX Angio Detect™) were negative.

On presentation the dog was depressed, but interested in its surroundings and able to stand and walk on its own. Initial vital signs included a rectal temperature of 38.1 °C, a heart rate of 120 beats/min, and a resting respiratory rate of 44 breaths/min. Reduced vesicular sounds were heard bilaterally on pulmonary auscultation. Mucous membranes were pale with a slightly prolonged capillary refill time. Emergency blood work was performed, and the CBC revealed a severe non-regenerative anemia with a hematocrit of 0.19 (see Table 1 for reference intervals). A mild thrombocytopenia with a platelet count of 182 × 10^9^/L was also noted. The leukogram and the biochemical profile (measuring alanine aminotransferase, alkaline phosphatase, gamma-glutamyl transferase, glucose, urea, creatinine, cholesterol, bilirubin, total protein, albumin, calcium, phosphate, sodium, and potassium) were unremarkable. C-reactive protein (CRP) was below the clinical decision limit applied at the laboratory (25 mg/L). Thoracic radiographs were performed, revealing moderate pleural effusion and areas of suspected consolidation. A coagulation panel and a TEG were requested, and the dog was taken directly to ultrasound for thoracic and abdominal evaluation in order to locate a focus for suspected hemorrhage. An ultrasound-guided diagnostic and therapeutic thoracocentesis was performed, revealing a hemorrhagic effusion (PCV 0.27). No underlying cause was detected. Abdominal ultrasound was unremarkable. The dog was hospitalized in an oxygen cage, providing 33% oxygen.

**Table 1:** Reference intervals for reported hematological and coagulation parameters

Hematocrit	0.39–0.59
Platelets	200–500 × 10^9^/L^a^
Fibrinogen	1–4 g/L
Eosinophils	0–1.2 × 10^9^/L
PT	Within 25% of reference pool^b^
aPTT	Within 25% of reference pool^c^
d-dimer	0–0.5 mg/L

*PT* prothrombin time; *aPTT* activated partial thromboplastin time

^a^Platelet counts were confirmed by evaluation of a blood smear unless otherwise stated

^b^PT measurements simultaneously performed on a healthy control pool: 7.7–9 s

^c^aPTT measurements simultaneously performed on a healthy control pool: 11.8–13.3 s

**Fig. 1:**
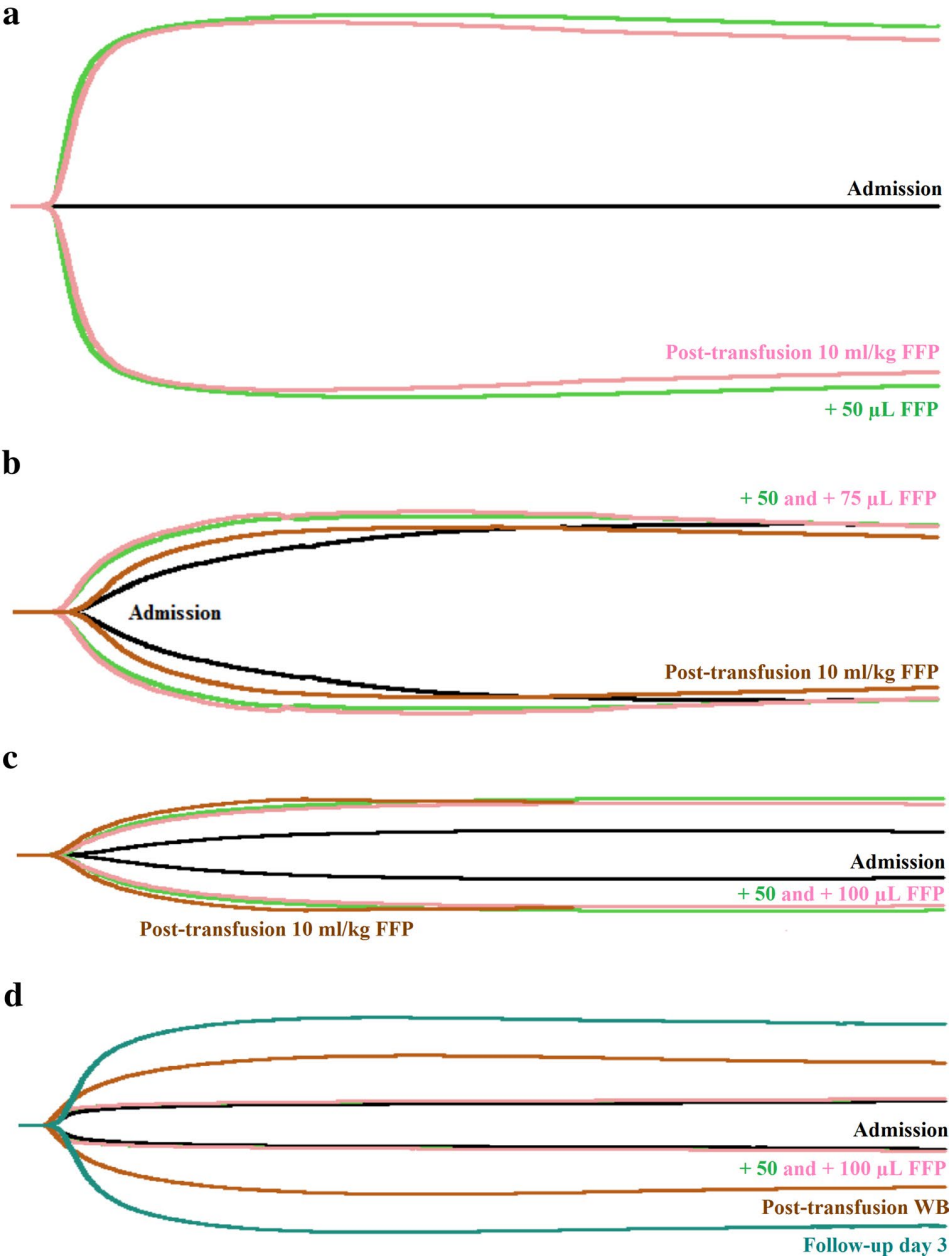
Thromboelastography-guided transfusion of four dogs with hypocoagulable disorders. **a** Dog with rodenticide poisoning (case 1): Thromboelastography (TEG) was performed at admission (black line) at which point the tracing flat-lined. A TEG with in vitro addition of 50 µL fresh frozen plasma (FFP-TEG), corresponding to a 10 mL/kg FFP transfusion, was shown to markedly improve the tracing (green line). Following a FFP transfusion of 10 mL/kg, a post-transfusion TEG (pink line) showed results similar to the FFP-TEG. **b** Dog with angiostrongylosis (case 2): TEG was performed at admission (black line) showing hypocoagulability. A FFP-TEG with addition of 50 µL FFP was shown to normalize R, K, and α and to improve MA (green line). A FFP-TEG with 75 µL FFP showed negligible additional improvement (pink line). Following transfusion with 10 mL/kg FFP, a post-transfusion TEG showed improved results (brown line), although not quite to the extent predicted in vitro, likely due to ongoing consumption of coagulation factors. **c** Dog with suspected disseminated intravascular coagulation (case 3): TEG was performed at admission (black line), showing severe hypocoagulability. A FFP-TEG with addition of 50 µL FFP was shown to improve, although not normalize all parameters of the TEG (green line). A FFP-TEG with 100 µL FFP showed no additional improvement (pink line). Following an FFP transfusion of 10 mL/kg, a post-transfusion TEG (brown line) showed results similar to the FFP-TEG. **d** Dog with primary immune-mediated thrombocytopenia (case 4): TEG was performed at admission (black line), showing severe hypocoagulability. A FFP-TEG with addition of 50 or 100 µL FFP did not cause any improvement (green and pink lines). The dog was provided with a fresh whole blood (WB) transfusion, and post-transfusion TEG revealed improvement, although not normalization of all TEG parameters (brown line). A follow-up TEG 3 days later revealed normalization of the tracing (blue line)

Results of the coagulation panel revealed an unmeasurably long PT (> 320 s), prolonged aPTT (33.4 s), unmeasurably low fibrinogen < 0.6 g/L, and a d-dimer of 0.3 mg/L. The TEG flat-lined (Fig. 1a), showing an infinite R corresponding to lack of clot formation. An FFP-TEG with the in vitro addition of 50 µL FFP, corresponding to a 10 mL/kg FFP transfusion, was performed. This resulted in a hypercoagulable tracing (Fig. 1a, Table 2), improving all TEG parameters, thus implying that transfusion of 10 mL/kg of FFP would be beneficial and sufficient. On further interview with the owner, it was discovered that the rodenticides floucoumafen and bromadiolone were continuously used at their home property. Hence, a tentative diagnosis of rodenticide intoxication was made.

**Table 2:** Thromboelastography data from four dogs with hypocoagulable hemostatic disorders

Parameter	R (min)	K (min)	α (degrees)	MA (mm)
Reference intervals	(3.0–9.0)	(2.0–8.0)	28.0–59.0	39.0–59.0
Case 1				
Admission TEG	> 120	Not reached	Not reached	Not reached
FFP-TEG (50 µL FFP)	5.7	1.3	71.5	75.2
Post-transfusion TEG	5.6	1.9	66.1	73.9
Case 2				
Admission TEG	10.1	15.6	20.2	27.2
FFP-TEG (50 µL FFP)	7.1	5.7	38.4	37.4
FFP-TEG (75 µL FFP)	7.0	4.8	41.2	39.0
Post-transfusion TEG	9.5	7.9	29.8	32.6
Case 3				
Admission TEG	11.8	Not reached	7.9	7.4
FFP-TEG (50 µL FFP)	7.3	15.8	23.8	22.3
FFP-TEG (100 µL FFP)	6.9	20.2	20.0	20.8
Post-transfusion TEG	6.3	11.7	28.9	25.7
Case 4				
Admission TEG	5.4	Not reached	27.1	8.7
FFP-TEG (50 µL FFP)	5.0	Not reached	37.4	9.8
FFP-TEG (100 µL FFP)	4.7	Not reached	40.2	10.0
Post-transfusion TEG (WB)	4.6	6.2	44.1	32.5
Follow-up TEG	5.6	2.8	54.8	53.5

Data is presented as baseline data (admission), investigation of the effect of fresh frozen plasma on the patient’s hemostatic state, and post-transfusion data

*TEG* thromboelastogram, *FFP* fresh frozen plasma, *WB* whole blood, *R* reaction time, *K* kinetics, *α* angle, *MA* maximal amplitude, *min* minutes, *mm* millimeters

Ten mL/kg of FFP was administered according to the amount predicted sufficient by the FFP-TEG, and the dog was started on vitamin K1 therapy (5 mg/kg). A TEG was repeated following the transfusion, showing a tracing corresponding to that predicted by the pre-treatment FFP-TEG (Fig. 1a, Table 2).

The following day the dog was bright, alert, and responsive. Mild tachypnea remained (44 breaths/min), but the dog did not deteriorate when taken out of the oxygen cage. On a repeated ultrasound examination, the amount of fluid in the pleural space was subjectively evaluated as similar to that seen the previous day following thoracocentesis. A CBC was repeated, revealing a hematocrit of 0.17, and a normalized platelet count (209 × 10^9^/L). A repeated TEG was normocoagulable. The dog was kept in the hospital for observation until the following day at which time it was clinically stable, and the hematocrit had increased to 0.24. The dog was discharged on vitamin K1 treatment for 5 weeks. Follow-up visits revealed a normalized hematocrit and a continually normal coagulation status as evidenced by normal TEG parameters.

### Case 2

A 1-year-old 27.5 kg intact male Vizsla presented with acute neurological signs characterized by ataxia and decreased awareness of its surroundings. The dog had been seen by its regular veterinarian the previous day due to depression and coughing, and the veterinarian had prescribed a combination of spot-on moxidectin and imidacloprid (Advocate© Bayer Animal Health) due to suspicion of angiostrongylosis.

At presentation the dog was in lateral recumbency. When encouraged to stand and walk, it was ataxic. Mentally, the dog was responsive, but star-gazing behavior and signs of left-sided hemineglect (decreased awareness of being approached from one side even though vision appeared to be present) were noted. Vital signs included a rectal temperature of 38.4 °C, a heart rate of 80 beats/min, and a respiratory rate of 24 breaths/min. Increased vesicular sounds were noted on pulmonary auscultation. Cutaneous petechiae were detected in the left groin and axilla. A mean arterial blood pressure of 94 mmHg was obtained.

Hematological, biochemical, and coagulation panels and a TEG were performed as were thoracic radiographs, a fecal smear, and the IDEXX Angio Detect™ test. A moderate non-regenerative anemia with a hematocrit of 0.28 and a moderate thrombocytopenia (119 × 10^9^/L) was found on the CBC. Biochemistry was unremarkable. CRP was moderately elevated. The coagulation panel revealed a mildly prolonged PT (12.8 s), a normal aPTT, a low fibrinogen (0.94 g/L), and an increased d-dimer (1.2 mg/L). The TEG (Fig. 1b, Table 2) was hypocoagulable (mildly prolonged R and K, low α and MA). A FFP-TEG with in vitro addition of 50 µL FFP resulted in normalizing of R, K, and α, with mild-moderate improvement in MA. An additional FFP-TEG with addition of 75 µL FFP (corresponding to 15 mL/kg FFP) showed negligible additional improvement of the MA. The antigen test as well as the fecal smear confirmed infection with *A. vasorum*. Thoracic radiographs revealed a generalized markedly increased interstitial pattern.

The dog was diagnosed with angiostrongylosis with a secondary coagulopathy, and it was suspected that its neurological signs were the result of a cerebral vascular event although larva migrans or a separate primary neurological problem could not be ruled out. A FFP transfusion of 10 mL/kg was administered, according to the amount predicted as optimal by the FFP-TEG, along with prednisolone (0.5 mg/kg SID for 3 days) for prevention of anaphylaxis following parasite death. After transfusion of the initial 10 mL/kg, another TEG was performed, revealing improvement on all parameters, but not to the extent seen in vitro, presumably due to ongoing pathology. Therefore, the transfusion was continued, however, following a total amount of 15 mL/kg, the dog developed a suspected mild transfusion reaction characterized by edema of the muzzle and palpebrae which responded to antihistamine treatment (mepyramine 1 mg/kg subcutaneously).

During the first 24 h ataxia resolved, and the dog seemed more mentally aware, but additional petechiae were noted in the groin and axilla, and more appeared over the course of the second day. A repeated TEG revealed a return to the state of the initial TEG. As the dog had developed more petechiae, it was concluded that additional transfusion therapy was needed. Due to the previous transfusion reaction, a different donor was chosen, and, also, cryoprecipitate treatment was decided upon in order to minimize the amount of plasma administered while still supplying coagulation factors, especially fibrinogen as the dog had presented with hypofibrinogenemia. Still, another similar mild transfusion reaction occurred following this transfusion. It was also decided to initiate fenbendazole treatment (25 mg/kg SID for 21 days) [8] although the dog had already received moxidectin/imidaclopride from its referring vet. The addition of fenbendazole was chosen because of the authors’ experience with and available evidence for this treatment protocol in the sub-group of *A. vasorum* positive dogs with secondary coagulopathies [9].

The following day the dog had improved further, but intermittent neurological signs were seen (similar to those at presentation). A repeated CBC revealed a hematocrit of 0.31, a platelet count of 175 × 10^9^/L, as well as mild eosinophilia (1.8 × 10^9^/L). A repeated TEG showed normalization of R, K, α, and MA, but with mildly increased LY30 and LY60 (percent fibrinolysis 30 and 60 min after MA), corresponding to mildly increased fibrinolysis. The dog was, therefore, kept in hospital for observation. The following day the dog was clinically unremarkable, no further petechiae were observed, PT and aPTT were normal, and the dog was discharged. The owner noted intermittent star-gazing behavior the first few days following discharge, and, therefore, the prednisolone treatment was prolonged for another week and then tapered. The dog continued to improve, and a Baermann fecal analysis performed 2 weeks after the end of treatment was negative.

### Case 3

A 10-year old 28.7 kg male neutered mixed-breed dog presented for a follow-up visit after having been hospitalized for 24 h the previous week with severe hemorrhagic gastroenteritis and hypovolemic shock. Prior to this episode the dog had been on 0.35 mg/kg oral prednisolone daily for months due to dermatological problems (described by the client as pruritus). This treatment had been stopped at the initial presentation with gastroenteritis. The dog’s coagulation status (PT, aPTT, TEG) had been unremarkable during hospitalization apart from a moderate thrombocytopenia (65 × 10^9^/L) which had not been confirmed microscopically. On presentation at the control visit the dog was bright, alert, and responsive, was eating and drinking normally, and the owner considered it to have recovered except for still having slightly soft stools. The dog was still receiving treatment with antibiotics and gastroprotectives.

The clinical exam was unremarkable. A hematological profile was analyzed, revealing a platelet count of 46 × 10^9^/L, a mild non-regenerative anemia (hematocrit 0.37) and mild eosinophilia (1.59 × 10^9^/L). Biochemistry was unremarkable. CRP was below 25 mg/L. A coagulation profile and a TEG were requested in order to further evaluate the patient’s hemostatic state and possible need for transfusion therapy, revealing an aPTT of 18 s, unmeasurable PT and fibrinogen, and a d-dimer of 3.7 mg/L. The TEG (Fig. 1c, Table 2) showed severe hypocoagulability (prolonged R, decreased α and MA; K was not reached). A FFP-TEG with the in vitro addition of 50 µL FFP was performed, leading to improvement of all parameters, but without complete normalization of the coagulation status. An additional FFP-TEG with 100 µL FFP (corresponding to 20 mL/kg FFP) did not result in further improvement. A von Willebrand factor analysis was also performed with a result of 68%. This assay was calibrated on a plasma pool from healthy dogs and the sample result extrapolated from the standard curve and reported as percentage with > 70% considered the normal range and < 50% considered abnormal.

It was considered most likely that the dog had developed disseminated intravascular coagulation (DIC) secondary to the severe systemic inflammatory reaction it had suffered the previous week. A secondary immune-mediated thrombocytopenia could not be ruled out. To rule out other underlying causes the dog was tested for tick-borne agents (serology for *Borrelia* sp., *Anaplasma* sp., and *Erhlichia* sp. measured with IDEXX 4dx) and gastrointestinal parasites (*Giardia* sp. and *Cryptosporidium* sp.), all of which were negative. A Baermann fecal analysis was performed, in which one larva, considered to be a *Crenosoma vulpis* larva, was found, but *A. vasorum* could not be completely ruled out due to the quality of the larva (an antigen test was not commercially available at this time). The dog had no history of exercise intolerance or coughing, and thoracic radiographs were unremarkable. As the dog had a history of skin problems which had previously responded to prednisolone, an antinuclear antibody test was also performed and was negative.

While the suspected DIC was subclinical at this stage, the hypocoagulability detected by TEG was considered of importance because it is a known negative prognostic factor for dogs with DIC [4]. Hence, as benefit of FFP transfusion was revealed by the FFP-TEG, this treatment was considered indicated. A transfusion of 10 mL/kg of FFP was administered, according to the amount predicted as optimal by the FFP-TEG. The dog was also started on fenbendazole in order to treat possible angiostrongylosis. A subsequent TEG revealed a tracing corresponding to the one predicted by the FFP-TEG. The dog remained clinically stable and was discharged. Final follow-up visits revealed normalization of all parameters of hemostasis.

### Case 4

An 8-year-old male 19.0 kg English Springer Spaniel presented with severe thrombocytopenia. Two months previously the dog had been diagnosed with a hepatopathy of unknown origin and a concurrent, possibly related, systemic inflammatory reaction. This had subsided on symptomatic treatment (antibiotics, antiemetics, and antioxidants), with almost total normalization of liver enzymes. The dog had been doing well since, but had developed a swelling on the chest following exercise the day before presentation. Antibiotics and antiemetics had been stopped 2 months and antioxidants 3 weeks prior to presentation.

At presentation the dog was quiet, alert, and responsive. Vital signs included a rectal temperature of 38.4 °C, a heart rate of 102 beats/min, and a respiratory rate of 32 breaths/min. Mucous membranes were pink and moist, but with evidence of petechiae on the gingiva. Cutaneous ecchymoses were similarly noted. The dog had a mean arterial pressure of 75 mmHg.

Hematological, biochemical, and coagulation panels as well as a TEG were requested. The dog was tested for *A. vasorum* on fecal smear and antigen test, both of which were negative. Serologic testing for *Erhlichia* sp., *Anaplasma* sp., and *Borrelia* sp. (IDEXX 4dx) was also performed and was negative. Abdominal ultrasound and thoracic radiographs were desired, but postponed as simply touching the dog was enough to induce further ecchymoses.

The CBC revealed a severe thrombocytopenia (8 × 10^9^/L) and was otherwise unremarkable, as was the biochemistry. CRP was below 25 mg/L. PT, aPTT, D-dimer, and fibrinogen were all unremarkable, but the TEG showed marked hypocoagulability (Fig. 1d, Table 2) characterized solely by a severely decreased MA. Performing FFP-TEG with in vitro addition of 50 and 100 µL FFP, respectively, did not lead to any change in the TEG tracing, leading to the conclusion that the bleeding disorder was caused simply by the severe thrombocytopenia without any complicating coagulopathy, and that supplying additional coagulation factors (including fibrinogen, an important contributor to the MA) would not benefit the patient. Instead the dog was blood-typed and a fresh whole blood transfusion administered in order to supply platelets. A platelet concentrate product would have been preferable as the dog was not lacking plasma or erythrocytes; however such a product was not available. Following the whole blood transfusion the platelet count had increased to 15 × 10^9^/L, and an increase in the TEG MA was seen. At this point abdominal ultrasound and thoracic radiographs were performed and were unremarkable.

The thrombocytopenia was considered to have a primary immune-mediated origin. No cause of a secondary immune-mediated reaction or increased platelet consumption had been found on the diagnostic work-up, but a bone marrow disorder leading to decreased megakaryocyte production could not be ruled out. A therapeutic trial with immunosuppressive doses of corticosteroids was attempted. Two days after initiation of treatment the platelet count had increased to 38 × 10^9^/L, with a simultaneous normalization of the TEG (Fig. 1d). As the dog was doing well and had not developed any further clinical signs of bleeding, it was discharged on prednisolone 2 mg/kg daily. The platelet count normalized within the next month, and steroid therapy was tapered.

## Discussion and conclusions

With the introduction of the cell-based model of hemostasis [10], viscoelastic assays such as TEG became widely used to deliver quantitative information on the entire hemostatic process including the clot formation, strength, life span, and break down. Unlike traditional assays they take into account both plasma and cellular elements of hemostasis. Their limitation in simulating hemostasis in vivo is mainly that they cannot detect the contribution of and abnormalities in the vascular endothelium, von Willebrand factor, platelet binding to the endothelium, or the shear forces of blood [7]. Hence, while the use of TEG may guide the clinician towards detection of hemostatic defects, it is important that the results be interpreted in the context of the patient’s clinical condition [11]. An additional limitation of viscoelastic assays involves the effect of erythrocytes, with some evidence to suggest that anemia confers a hypercoagulability to the assay [12]. As the patients described in this case series were all hypocoagulable, that is of less importance in the present context.

In human medicine TEG, or another viscoelastic assay known as the ROTEM (rotational thromboelastometry), are frequently used to guide transfusion therapy in trauma-induced coagulopathy [6]. Applying TEG along with transfusion algorithms has been shown to reduce transfusion requirements in trauma care [7]. Similarly, TEG-guided transfusion in post-operative care is frequently used. A normal TEG in the context of post-surgical bleeding thus indicates the need for surgical re-exploration of the site rather than transfusion therapy [7, 13]. In human medicine, applying the viscoelastic assays in this way allows for precision medicine with the right blood product (FFP, packed RBCs, platelet concentrate) administered in the right amount at the right time, minimizing the risk of adverse effects of transfusions, and optimizing the ethical use of blood products by limiting unnecessary use. The success of these guidelines in human medicine might inspire investigation into similar application for veterinary patients.

The cases reviewed here represent a variety of hypocoagulable disorders in dogs and the use of TEG to achieve targeted replacement therapy of the individual patient. This use of TEG has not been previously reported in dogs, to the best knowledge of the authors. In the set-up reported here, TF was applied as the activator. This is the standard activator used at the laboratory as it most closely simulates the in vivo activation of coagulation. As kaolin-TEG is used for human TEG-guided transfusion guidelines [6] it is possible that kaolin-activated FFP-TEG would work for dogs as well, but future studies are required for confirmation.

The first case was a dog with rodenticide poisoning, known to affect the vitamin K-dependent coagulation factors by antagonizing vitamin K, thus rendering the liver unable to activate factors II, VII, IX, and X, ultimately resulting in bleeding [14]. While it is well-established that dogs with hemorrhage due to rodenticide poisoning benefit from FFP transfusions, TEG can play a greater role in these cases than simply affirming this fact. The in vitro addition of FFP to the analysis allows a quantitative interpretation, hence evaluating the dose of plasma needed to achieve a normocoagulable state. In patients at increased risk of volume overload this has the added benefit of being able to provide as little colloid as necessary.

The second case presented with *A. vasorum* infection, a parasite known to cause hemorrhage in up to a third of affected patients [15]. The pathophysiological mechanism in this case has not been clearly determined, but a low-grade, chronic DIC has been suspected, leading to depletion of both coagulation factors and platelets [15]. A recent study has revealed that a common cause of bleeding in these dogs may be hyperfibrinolysis [16], a hemostatic disorder which cannot be detected on standard coagulation testing, making TEG all the more useful to investigate the cause of bleeding and guide therapy in such animals. The dog in the present case did not have signs of hyperfibrinolysis on its baseline TEG, but did develop a mild hyperfibrinolysis during its hospitalization.

The third case was a dog with suspected DIC, a complex hemostatic disorder in which an imbalance exists between the pro- and anticoagulant systems [4], and for which treatment is not straight-forward. In this specific case the dog did not have clinical signs of bleeding at the time of presentation, but was considered at high risk of deterioration due to its worsening thrombocytopenia and recent severe inflammatory disease. It is possible that this dog would have recovered without transfusion therapy. However, hypocoagulability as detected by TEG is a poor prognostic factor in dogs with DIC [4], and, as the FFP-TEG analysis supported a benefit of administering FFP to this dog, transfusion therapy was, therefore, considered a beneficial approach.

The final case represented a pure platelet-related disorder in which no benefit of administrating FFP could be detected. These dogs are a challenge in veterinary medicine as we generally do not have platelet concentrates available. Thromboelastography was applied in this case to investigate whether additional hemostatic complications had occurred and whether supplying FFP with its supply of especially fibrinogen would be of benefit in optimizing clot strength. The assay revealed no benefit of FFP, and a fresh whole blood transfusion was, therefore, the only way of optimizing the dog’s hemostatic status by providing fresh platelets. Once the platelet count had increased, albeit minimally, no further bleeding was detected, and the effect of immunosuppression had time to prevail.

In conclusion, this retrospective case-series indicates that the ability of TEG analysis to assist in providing targeted therapy and monitoring the bleeding risk of dogs with hypocoagulable disorders could be of benefit in the emergency setting. Larger prospective studies are required to evaluate whether such targeted therapy reduces the morbidity and mortality associated with bleeding disorders, and whether algorithms similar to those currently used in human patients are of value in guiding veterinary transfusion therapy [6].

## Data Availability

All data generated or analyzed during this study are included in this published article.

## Abbreviations

α: angle
aPTT: activated partial thromboplastin time
CRP: C-reactive protein
DIC: disseminated intravascular coagulation
FFP: fresh frozen plasma
FFP-TEG: thromboelastography with in vitro supplementation of FFP
K: kinetics
MA: maximal amplitude
PT: prothrombin time
R: reaction time
TEG: thromboelastography
TF: tissue factor
